# Recurrence-Free Survival in Early and Locally Advanced Large Cell Neuroendocrine Carcinoma of the Lung after Complete Tumor Resection

**DOI:** 10.3390/jpm13020330

**Published:** 2023-02-15

**Authors:** Barbara Altieri, Anna La Salvia, Roberta Modica, Francesca Marciello, Olaf Mercier, Pier Luigi Filosso, Bertrand Richard de Latour, Dario Giuffrida, Severo Campione, Gianluca Guggino, Elie Fadel, Mauro Papotti, Annamaria Colao, Jean-Yves Scoazec, Eric Baudin, Antongiulio Faggiano

**Affiliations:** 1 Division of Endocrinology and Diabetes, Department of Internal Medicine I, University Hospital, University of Würzburg, 97080 Würzburg, Germany; 2 National Center for Drug Research and Evaluation, National Institute of Health (ISS), 00161 Rome, Italy; 3 Department of Clinical Medicine and Surgery, Federico II University, 80131 Naples, Italy; 4 Department of Thoracic Surgery and Heart and Lung Transplantation, Université Paris-Saclay, Marie Lannelongue Hospital, GHPSJ, 92350 Le Plessis Robinson, France; 5 Department of Thoracic Surgery, University of Turin, San Giovanni Battista Hospital, 10126 Turin, Italy; 6 Department of Thoracic and Cardiovascular Surgery, University Hospital Rennes Pontchaillou, University of Rennes, 422931 Rennes, France; 7 Clinical Oncology Unit, Department of Experimental Oncology, Mediterranean Institute of Oncology, 95029 Catania, Italy; 8 Department of Advanced Technology, Pathology Unit, Cardarelli Hospital, 80131 Naples, Italy; 9 Department of Thoracic Surgery, Cardarelli Hospital, 80131 Naples, Italy; 10 Department of Oncology, Pathology Unit, University of Turin, 10126 Turin, Italy; 11 Department of Pathology, Institute Gustave Roussy, Université Paris Saclay, 94805 Villejuif, France; 12 Endocrine Oncology and Nuclear Medicine Department, Institute Gustave Roussy, Paris-Saclay University, 94805 Villejuif, France; 13 Endocrinology Unit, Department of Clinical and Molecular Medicine, Sant’Andrea Hospital, Sapienza University of Rome, 00189 Rome, Italy

**Keywords:** neuroendocrine tumor, LCNEC, pulmonary cancer, prognostic marker, prognosis, survival, lymph nodes, age, surgery, adjuvant therapy

## Abstract

Background: Large Cell Neuroendocrine Carcinoma (LCNEC) is a rare subtype of lung cancer with poor clinical outcomes. Data on recurrence-free survival (RFS) in early and locally advanced pure LCNEC after complete resection (R0) are lacking. This study aims to evaluate clinical outcomes in this subgroup of patients and to identify potential prognostic markers. Methods: Retrospective multicenter study including patients with pure LCNEC stage I-III and R0 resection. Clinicopathological characteristics, RFS, and disease-specific survival (DSS) were evaluated. Univariate and multivariate analyses were performed. Results: 39 patients (M:F = 26:13), with a median age of 64 years (44–83), were included. Lobectomy (69.2%), bilobectomy (5.1%), pneumonectomy (18%), and wedge resection (7.7%) were performed mostly associated with lymphadenectomy. Adjuvant therapy included platinum-based chemotherapy and/or radiotherapy in 58.9% of cases. After a median follow-up of 44 (4–169) months, the median RFS was 39 months with 1-, 2- and 5-year RFS rates of 60.0%, 54.6%, and 44.9%, respectively. Median DSS was 72 months with a 1-, 2- and 5-year rate of 86.8, 75.9, and 57.4%, respectively. At multivariate analysis, age (cut-off 65 years old) and pN status were independent prognostic factors for both RFS (HR = 4.19, 95%CI = 1.46–12.07, *p* = 0.008 and HR = 13.56, 95%CI 2.45–74.89, *p* = 0.003, respectively) and DSS (HR = 9.30, 95%CI 2.23–38.83, *p* = 0.002 and HR = 11.88, 95%CI 2.28–61.84, *p* = 0.003, respectively). Conclusion: After R0 resection of LCNEC, half of the patients recurred mostly within the first two years of follow-up. Age and lymph node metastasis could help to stratify patients for adjuvant therapy.

## 1. Introduction

Large cell neuroendocrine carcinoma (LCNEC) of the lung represents a rare subtype of primary lung cancer, accounting for approximately 3% of cases [1,2]. Recent evidence suggests that the incidence of LCNEC is slowly increasing by 0.011 people per 100,000 per year, especially for cases with metastatic disease at diagnosis [3]. According to the 2021 World Health Organization (WHO) classification of thoracic tumors, LCNEC, together with small cell lung carcinoma (SCLC), is a subtype of high-grade, poorly differentiated carcinoma demonstrating neuroendocrine features [4]. The demonstration of the neuroendocrine nature of the tumor requires the identification of one or more neuroendocrine markers (synaptophysin, chromogranin A, CD56, or INSM1) in at least 10% of tumor cells [4]. 

The prognosis of LCNEC is poor, with a 5-year overall survival (OS) rate ranging from 39–53% in a cohort including mainly early tumor stages [5,6,7,8,9] and decreasing up to 15–20% in advanced stage [7,10], mirroring survival trend of SCLC [2,3]. 

Due to the rarity of LCNEC and because most of them are diagnosed in the advanced stage [3], there is a lack of prospective studies or strong evidence to guide treatment in early-stage disease, which is often extrapolated from both non-small-cell lung cancer (NSCLC) and SCLC guidelines [11,12,13]. As suggested for NSCLC, a complete tumor resection associated with mediastinal lymph node dissection should be considered for early-stage LCNEC patients in the context of a multimodal treatment concept [11,12]. In patients with early-stage SCLC, surgery may be considered in patients with clinical stages I and II (cT1–2N0) in the context of a multimodal treatment concept and following a multidisciplinary board decision [III, B] [13]. Two large population-based studies in LCNEC evaluating the Surveillance, Epidemiology, and End Results (SEER) database showed that surgical resection of the tumor was significantly associated with a better OS compared to not resected tumor, independently to tumor stage [3,14]. However, in addition, the early-stage disease rapidly recurs after the tumor resection, with a 5-year recurrence-free survival (RFS) of 35–43% [8,9,15,16]. Therefore, adjuvant/multimodal treatment is commonly administered, similarly to the treatment of SCLC, showing potential better survival even in stage I disease [6,7,17,18,19]. A small single-arm prospective trial demonstrated that adjuvant cisplatin-etoposide chemotherapy was associated with a significant OS improvement compared to historical data of patients treated with surgery alone [20]. However, some studies failed to find a clinical improvement in patients treated with adjuvant chemotherapy and/or radiotherapy [9,14,16,21]. 

Although the 5-year rate of recurrence in LCNEC (also at early-stage) is high and similar or close to the 5-year OS rate [8,9,10,16], most studies, including large national databases [3,6,14,19], focused as the primary endpoint to OS. Moreover, data on RFS in early-stage LCNEC are lacking [8,9,16,20]. However, all these above-mentioned studies also included patients with not-complete LCNEC resection, histological diagnosis of mixed type of LCNEC, as well a very small percentage of patients with advanced diseases. Only four studies, summarized in Table 1, evaluated patients with early and locally advanced LCNEC after complete tumor resection (R0) and with histological diagnosis of pure LCNEC [21,22,23,24]. 

Particularly, one study reported a median RFS of 49 months [23]. The other three studies showed a 5-year OS rate ranging between 47% and 51% [21,22,24]. Therefore, a need emerges for a new challenge and refocus the endpoint on recurrence. In this study, we assessed the primary endpoint of the RFS in patients with early and locally advanced pure LCNEC after complete resection. The secondary aims were the evaluation of the disease-free survival and potential prognostic markers of clinical outcome.

## 2. Materials and Methods

### 2.1. Study Design and Population

We retrospectively collected data from consecutive patients from November 1997 to December 2014 with a diagnosis of LCNEC coming from 3 Italian (“Federico II” University Hospital and “Antonio Cardarelli” Hospital, Naples; University of Torino, Torino; Mediterranean Institute of Oncology, Catania) and 3 French (Institute Gustave Roussy, Villejuif; Marie Lannelongue Hospital, Paris-Sud University, Le Plessis Robinson; University Hospital Rennes Pontchaillou, University of Rennes, Rennes) European neuroendocrine tumor society (ENETS), European Reference Network (ERN) or national expert centers. The last follow-up was in December 2020. Only patients with early or locally advanced LCNEC (tumor stage I-III according to the International Association for the Study of Lung Cancer (IASLC) tumor, node, and metastasis (TNM) classification [25]) and with R0 resection, considered as microscopically margin-negative resection [26], were included in this study. All surgically resected specimens were independently reviewed by three expert pathologists (M.P. and S.C. for the Italian specimens and J.-Y.S. for the French specimens) to confirm the diagnosis of LCNEC according to the WHO criteria for lung NEC [4]. Patients with mixed histology having additional cell subtypes (combined LCNEC) after the histological revision were excluded from the study. Other exclusion criteria were: the presence of tumor metastasis at the diagnosis and not completely microscopically or macroscopically resected tumor. Moreover, patients who underwent preoperative induction chemotherapy and/or radiotherapy were also excluded.

Work-up for diagnosis and staging was performed following European Society for Medical Oncology (ESMO) clinical practice guidelines for NSCLC [12]. The extension of tumor resection as well as the administration of adjuvant therapy was determined by the institution’s multidisciplinary board decision according to tumor staging and risk assessment. During follow-up, all patients underwent standardized follow-up every six months with history and physical examination, and imaging procedures, including computed tomography (CT) of the chest and/or abdomen and magnetic resonance imaging (MRI) of the brain or whole body ^18^F-fluorodeoxyglucose positron emission tomography/CT (^18^FDG-PET/CT) every 6–12 months according to risk stratification [12] and institution multidisciplinary board decision.

Clinical characteristics, including age, sex, anthropometric measurements (i.e., height and weight), symptoms at diagnosis, smoking habits, comorbidities, tumor size, imaging procedures, type of surgical resection, assessment of lymph nodes metastasis, pathological tumor stage, adjuvant treatment, as well as follow-up and patient outcome were collected from medical records. Body mass index (BMI) was evaluated from the anthropometric measurements and was classified according to the WHO criteria as normal weight (BMI 18.5–24.9 kg/m^2^), overweight (BMI 25.0–29.9 kg/m^2^), and obesity (BMI ≥ 30.0 kg/m^2^), as previously reported [27,28,29]. Symptoms at diagnosis were considered tumor mass-related or hormonal-related. Clinically asymptomatic tumors were diagnosed incidentally. The presence of comorbidities, including type 2 diabetes mellitus, hypertension, and cardiovascular events (i.e., acute myocardial infarction, angina, stroke, and transient ischemic attack) were also collected. Smoking status was evaluated as ‘smoker’, in the case of former or current smokers, and ‘non-smoker’, as previously reported [29,30]. Tumor size with a cut-off of 3 cm, which was previously reported to predict the clinical outcome significantly [10,21], was considered in the analysis. The type of surgical resection was analyzed as ‘wedge resection/lobectomy’ and ‘pneumectomy/bilobectomy’, as previously reported [7]. Type of adjuvant treatment after the R0 resection was analyzed in three groups ‘chemotherapy or radiotherapy alone’, ‘chemotherapy + radiotherapy’, and ‘no adjuvant treatment’. Clinical outcome, evaluated both in terms of recurrence-free survival (RFS) and disease-specific survival (DSS), was analyzed in relation to all these variables. 

This study was conducted in accordance with the Declaration of Helsinki and was approved by the local Ethics Committees of all centers. Written informed consent was obtained from all included patients.

### 2.2. Statistical Analysis

Continuous variables were expressed as median with range, whereas categorical variables as numbers and percentages. Categorical variables were compared using Fisher’s exact test or the Chi-square (χ^2^) test, as appropriate. RFS was defined as the time from surgery to the first radiological evidence of tumor relapse or alive at final follow-up. DSS was defined as the time from tumor resection to disease-related death or the end of data collection. OS was defined as the time from tumor resection to any cause of death or the end of data collection. Cumulative survival and difference were analyzed by Kaplan–Meier method and log-rank test. Cox proportional hazards regression model was performed to assess risk factors in the univariate and multivariate analyses. A hazard ratio (HR) with a 95% confidence interval (CI) was also considered. Due to the low number of events, only parameters with a *p*-value less than 0.20 on univariate regression analysis were selected for the multivariable model. A *p*-value < 0.05 was considered statistically significant. 

Statistical analysis was performed using SPSS Software (PASW Version 21.0, SPSS Inc., Chicago, IL, USA) and GraphPad Prism (version 5.0, La Jolla, CA, USA).

## 3. Results

### 3.1. Study Population and Treatment

A total of 45 subjects with a first diagnosis of early or locally advanced (stage I-III) completed resected LCNEC were enrolled. After pathological revision, six patients with combined LCNEC/SCLC were excluded from the study. Therefore, a final number of 39 patients (13 women and 26 men, median age at diagnosis 64 years, range 44–83 years) was enrolled. The clinical and pathological characteristics, as well as the type of surgery and adjuvant treatment, are summarized in Table 2. 

A normal BMI was reported in 53.8% of cases. Most of the patients (87.2%) were current or former smokers. Seventeen (43.8%) patients reported comorbidities. The most common clinical presentation at diagnosis was associated with tumor-related symptoms (66.7% of cases), among which fever was the most frequent, whereas the tumor was incidentally discovered in 13 (33.3%) patients. 

The median tumor size was 5.0 (1.3–18.0) cm, and 87.2% of patients had a tumor with a diameter larger than 3 cm. Pleural invasion was observed in 13 (33.3%) patients.

All patients underwent thoracic CT with complete pre-operative staging. Particularly thoracoabdominal CT was performed on 37 (94.9%) patients, whereas the two patients who had not undergone abdominal CT were evaluated by abdominal ultrasound. Contrast-enhanced CT of the brain was performed in 15 (38.5%) cases. Preoperative whole body ^18^FDG-PET/CT was performed on 15 (38.5%) patients, with positive results in 14 (93.3%) cases, whereas ^111^In-pentetreotide scintigraphy (somatostatin receptor scintigraphy, SRS) had been performed in 2 (5.1%) patients, with positive results in both. Thirteen (33.3%) patients underwent preoperative bone scintigraphy, including nine patients who had not undergone ^18^FDG-PET. Preoperative bronchial microscopy was performed in 22 (56.4%) patients. Contributive results were obtained for 12 (54.5%) cases.

All patients underwent a complete (R0) tumor resection. The most used surgical procedure was lobectomy (69.2%), associated with lymph node dissection in 92.5% of cases (Table 2). Postoperative complications (including pneumonia, air leak from thoracotomy tubes for more than seven days postoperatively, lobar collapse on postoperative chest radiography, empyema, and arrhythmia) occurred in thirteen (33.3%) cases, leading to one (2.5%) postoperative death. 

**Figure 1:**
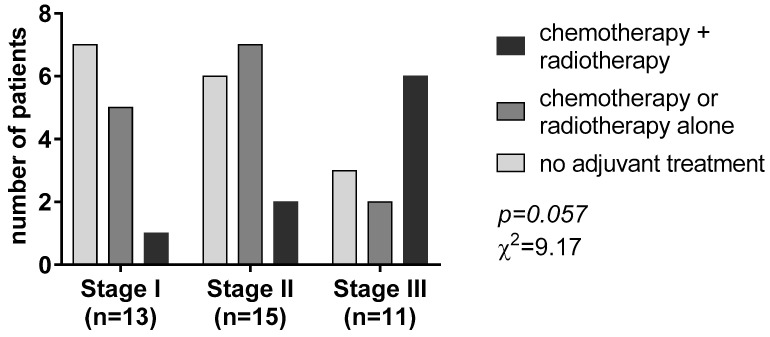
Adjuvant treatment according to the tumor-nodal-metastasis (TNM) staging system.

According to the postoperative pathological TNM system, tumor stage I, II, and III tumors accounted for 33.3%, 38.5%, and 28.2% of the patients, respectively. After R0 resection of the primary, adjuvant therapy, including platinum-based chemotherapy and/or radiotherapy, was given to 24 (61.5%) patients according to the tumor stage (χ^2^ = 9.17, *p* = 0.057, Figure 1). 

Most patients with stage I disease (53.8%) did not receive any adjuvant treatment. Chemotherapy or radiotherapy alone was the most given adjuvant treatment in patients with stage II disease (46.8%). On the contrary, most patients with tumor stage III (n = 6, 54.5%) received chemotherapy associated with radiotherapy (Figure 1).

### 3.2. Clinical Outcome

After a median follow-up of 44 (4–169) months, 21 (46.2%) patients experienced tumor relapse, and 17 (43.6%) died because of the disease. First tumor relapse was slightly more frequent at the locoregional level (12 cases, 57.1%) than distant metastasis (9 cases, 42.9%, including two brain metastases and one case of bone, liver, neck lymph nodes and thyroid, respectively, and not specified in two patients). 

**Figure 2:**
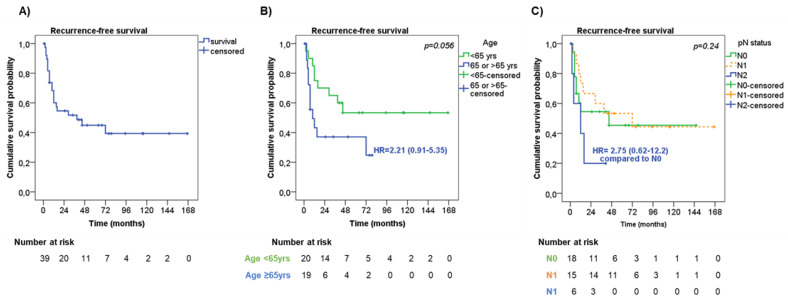
Recurrence-free survival. (**A**) Recurrence-free survival (RFS) in the entire cohort of patients. RFS according to age (**B**) and lymph node metastasis—pN status (**C**).

Median time to RFS was 39 months with 1-, 2- and 5-year RFS rates of 60.0%, 54.6%, and 44.9%, respectively (Figure 2A). 

At univariate analysis (Table 3), men showed a slightly shorter RFS compared with women (median RFS 29 months vs. ‘not reached’, respectively, HR = 1.77, 95%CI = 0.71–4.40, *p* = 0.22).

Also, older patients (≥65 years old) had a trend to a shorter RFS in comparison with younger patients (median RFS 10 months vs. ‘not reached’, respectively, HR = 2.21, 95%CI = 0.91–5.35, *p* = 0.056, Figure 2B). Among the pathological characteristics, the presence of pleural invasion had a trend to a shorter RFS than those without (median RFS 12 months vs. 72 months, respectively, HR 1.76 95%CI = 0.66–4.70, *p* = 0.19). No significant difference in RFS was observed according to tumor stage (*p* = 0.63). Considering the pathological assessment of lymph nodes metastasis (pN), patients with pN2 had a higher risk of a shorter RFS (median RFS 12 months) compared with pN0 and pN1 patients (median RFS not reached and 72 months, respectively, *p* = 0.24, Figure 2C). Patients who underwent wedge resection/lobectomy had a trend to a shorter RFS than those who underwent bilobectomy/pneumectomy (median RFS 16 months vs. ‘not reached’, respectively, HR = 2.09, 95%CI = 0.79–5.52, *p* = 0.14), particularly when considering patients with tumor stage III (HR = 4.64, 95%CI = 0.99–21.79, *p* = 0.051). Adjuvant therapy was not significantly associated with RFS (*p* = 0.21). However, by stratifying patients according to tumor stage, adjuvant treatment slightly correlated with RFS (*p* = 0.047). To note, patients with tumor stage III who did not receive adjuvant treatment or who were treated with chemotherapy or radiotherapy alone had a shorter RFS (median RFS 9 months, HR = 7.67, 95%CI = 0.62–94.57, and 7.5 months, HR = 10.95, 95%CI = 0.75–160.80, respectively) than those treated with chemo- and radiotherapy (median RFS 72 months). 

At multivariate analysis, age at diagnosis (HR = 4.19, 95%CI = 1.46–12.07, *p* = 0.008 for older patients) and pN status (HR = 13.56, 95%CI = 2.45–74.89, *p* = 0.003 for pN2) were found to be independent risk factors for RFS (Table 3). 

**Figure 3:**
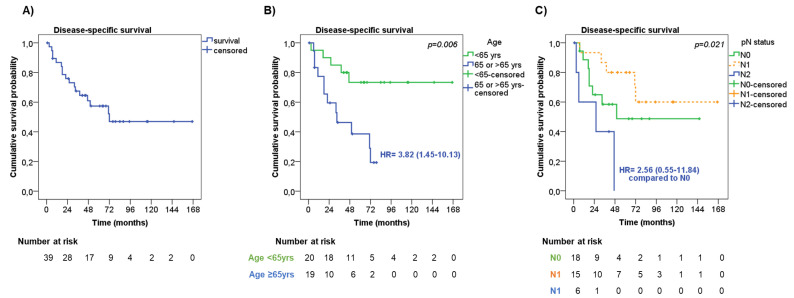
Disease-specific survival. (**A**) Disease-specific survival (DDS) in the entire cohort of patients. DSS according to age (**B**) and lymph node metastasis—pN status (**C**).

The median time of DSS was 72 months, with 1-, 2- and 5-year DSS rates of 86.8%, 75.9%, and 57.4%, respectively (Figure 3A). Seven patients died from other causes during the follow-up, leading to a median OS of 51 months, with 1-, 2- and 5-year OS rates of 82.0%, 69.2%, and 47.0%, respectively. 

At univariate analysis (Table 4), men showed a slightly shorter DSS than women (median DSS 50 months vs. ‘not reached’, respectively, HR = 1.69, 95%CI = 0.62–4.59, *p* = 0.30).

Older patients had significantly shorter DSS compared with younger patients (median DSS 33 months vs. ‘not reached’, respectively, HR = 3.82, 95%CI = 1.45–10.13, *p* = 0.006, Figure 3B). The presence of pleural invasion was slightly associated with a shorter DSS compared with those without (median DSS 33 months vs. ‘not reached’, respectively, HR = 1.78, 95%CI = 0.62–5.11, *p* = 0.23). No significant difference in DSS was observed considering the tumor stage (*p* = 0.29) and tumor size (*p* = 0.84). On the contrary, the pN status was significantly associated with DSS (*p* = 0.021), having patients with pN2 had a shorter DSS (median DSS 26 months) compared with pN0 and pN1 patients (median DSS 50 months and ‘not reached’, respectively, Figure 3C). Patients who underwent wedge resection/lobectomy had an increased risk of shorter DSS than those who underwent bilobectomy/pneumectomy (median DSS 71 months vs. ‘not reached’, respectively, HR = 2.07, 95%CI = 0.69–6.20, *p* = 0.19), and this was more evident in patients with tumor stage III (HR = 3.94, 95%CI = 0.87–17.80, *p* = 0.07). The type of adjuvant therapy was not significantly associated with DSS (*p* = 0.66), also by stratifying the patients according to tumor stage (*p* = 0.30).

Age at diagnosis (HR = 9.30, 95%CI 2.23–38.83, *p* = 0.002 for older patients) and pN status diagnosis (HR = 11.88, 95%CI = 2.28–61.84, *p* = 0.003 for pN2) were found to be independent risk factors of DSS both at univariate and multivariate analysis, including the above-mentioned parameters (Table 4). 

## 4. Discussion

LCNEC is a rare subtype of lung cancer with a high relapse rate [31]. Due to its low frequency, data related to early-stage LCNEC derived only from a relatively low number of studies [6,7,9,14,15,16,19], which included heterogeneous cohorts of patients having also patients with advanced diseases or who did not undergo tumor resection, or with a diagnosis of combined-LCNEC at histopathological analysis. Studies including only early and locally advanced LCNEC after complete resection of the tumor are very few [21,22,23,24] and focused mostly on overall survival (Table 1). Only the study by Zhang et al. [23] reported a median RFS of 49.3 months. In addition, median follow-up ranged between 24.9 and 47 months in only two studies [21,22] (Table 1).

The study by Casali et al. [22], as well as that by Zhang et al. [23], failed to find prognostic markers of RFS. Casali et al. showed that c-kit protein-positive immunostaining represented a negative prognostic factor of OS [22]. However, this result was not confirmed by multivariate analysis. The study by Zhang et al. demonstrated that patients with serum albumin levels below the normal range (≤35 g/L) had significantly worse survival compared to those having serum albumin within the normal range [23]. However, none of these parameters was further validated. The remaining two studies [21,24] were more successful in finding potential prognostic factors (Table 4). However, the higher rate of tumor relapse (range 45.4–55.7% of cases) observed in this selected subgroup of early stage LCNEC after R0 resection [21,22,23,24] stressing the importance of the validation of clear prognostic factors that could help clinicians to better stratify early stage LCNEC patients and to optimize their management after complete resection. Therefore, the analysis of recurrence free survival and specific prognostic factors represents an urgent unmet need. Our multicentric study, suggests potential cure of half of LCNEC patients after for the first time of median follow-up of more than 3 years.

In keeping with previous studies, we confirmed that LCNECs are often associated with the male sex (67% older age, median age 64 years) and smoker status (87%) [6,7,19,21,24]. However, in some other studies, a prevalence of females or non-smokers was reported [9,14,24]. Most patients reported non-specific symptoms, such as fever or chest and back pain, whereas cough and hemoptysis were only described in a few patients (18%), in line with previous data [1,32]. 

In this study, we confirm that the prognosis of early-stage LCNEC patients after R0 resection is poor, with a 5-year DSS rate of 57.4%, similar to previous studies [21,22,23,24]. Tumor recurrences were observed mostly within the first two years of follow-up, and the first recurrence was more frequently found in intrapulmonary, followed by brain metastases. Different from our study, bone was reported to be a frequent site of recurrence in LCNEC [15,21,24]. This discrepancy could be related to the fact that we evaluated the site of the first tumor relapse only, as well as to the sensitivity of different imaging procedures [33]. However, the high frequency of recurrence after the complete tumor resection implies a frequent follow-up with total-body imaging. 

Treatment regimens used in LCNEC are variable. According to the current ESMO clinical practice guidelines for NSCLC, the treatment of potentially resectable early-stage lung cancer (stage I and II) is the surgical removal of the tumor [12]. Particularly, anatomic pulmonary resection (e.g., segmentectomy, lobectomy, bilobectomy, pneumonectomy) and lymph node resection over wedge resection have been suggested because of the decreased risk of tumor relapse. However, a clear prognostic value for the type of surgery in early-stage LCNEC has not been elucidated. Some evidence supports that a less radical surgery significantly increased the clinical outcome [7,34]. On the contrary, other works report better outcomes for patients who received lobectomy compared to the ones that received sub-lobar resections [14]. In our analysis, bilobectomy/pneumectomy was associated with slightly improved RFS and DSS compared to wedge resection/lobectomy, and this was particularly evident in patients with tumor stage III.

Regarding systemic treatment, a huge amount of data supported the efficacy of adjuvant chemotherapy after surgery for LCNEC, even in the early stage [6,7,15,17,19,21]. In our experience, adjuvant therapies did not improve RFS and DSS compared to surgery alone. Only in patients with tumor stage III the association of chemo- and radiotherapy showed a slightly better clinical outcome in terms of RFS in comparison to chemotherapy or radiotherapy alone or the absence of adjuvant treatment. This result could be explained by the use of adjuvant chemotherapy and/or radiotherapy, predominantly in patients with tumor stage II-III. At the same time, surgical resection alone was predominantly chosen in patients with tumor stage I, as previously reported [21] and in line with ESMO guidelines on NSCLC [12]. 

Tumor stage at diagnosis has been identified as an independent prognostic factor influencing patient outcomes also for LCNEC [7,10,24,32] but failed to represent a prognostic marker after R0 tumor resection (Table 4) [21,22,23,24]. However, considering T and N stages separately, both factors had an impact on RFS and OS (Table 1) [21,24]. T stage, as well as tumor size with a 3 cm cut-off, were found to be prognostic factors for RFS and OS in two different cohorts of early stage-R0 LCNEC [21,24], although we were not able to confirm this result. On the contrary, considering our cohort and the study by Shen et al. [24], pN was confirmed to be an independent predictor of RFS, with N2 tumors having a significantly shorter RFS (median RFS 12 months in N2 in both studies) compared to N0-1 tumors. Moreover, in our study, N2 status was also found to be an independent prognostic factor for DSS. A role for the nodal status has also been described in studies including all tumor stages of LCNEC [10,32]

Interestingly, we identified a relevant role for patient age, with older patients having a significantly higher risk of tumor recurrence and death. A previous analysis detected age (≤64 vs. >64 years) as an independent factor influencing OS for LCNEC patients, with an increased risk of death in older patients, twice as higher compared to younger patients [34]. Recently, a nomogram model has been developed to predict the survival probability of LCNEC patients [35]. In this study, the data of 3076 LCNEC cases coming from the SEER registry were included, and age, categorized subjectively as ≤60, 60–70, and >70 years, was an independent predictor of survival [35].

The limit of this study is mostly represented by its retrospective nature, the cross-sectional experimental design, and the low number of patients that, although related to the rare incidence of LCNEC, could influence the interpretation of the results. Moreover, in the evaluation of the clinical outcomes, patients were considered together independently from the tumor stage. Therefore, the reported results should be viewed with caution, and a larger population should be investigated to attain an appropriate conclusion. Another limitation of the study is that the extension of pulmonary resection and adjuvant therapy was determined by local tumor board discussions, which could create some bias in the results. However, our study also has some strengths: (1) we included a well-selected cohort of LCNEC patients with stringent inclusion/exclusion criteria, restricted to tumor stage I-III, R0 resection, and pure LCNEC at histological diagnosis; (2) a centralized pathology review process allowed us to exclude patients with combined LCNEC/SCLC tumor; (3) we assessed for the first time the RFS as the primary endpoint.

## 5. Conclusions

Nodal status was identified as the most relevant prognostic factor. Moreover, we have found an independent prognostic value for patients’ age, both in terms of RFS and DSS, as well. Adjuvant trials stratified on the N status and age are needed. 

## Figures and Tables

**Table 1:** Review of the literature on clinical outcomes in early and locally advanced pure LCNEC after complete tumor resection compared to our study.

Parameter	Casali et al., Ann Thorac Surg. 2004 [22]	Zhang et al., Onco Targets Ther. 2015 [23]	Cattoni et al., World J Surg. 2019 [21]	Shen et al., Front Oncol. 2020 [24]	Current Study
Patients, n	33	50	72	94	39
Male, n (%)	31 (93.9)	47 (94.0)	43 (59.7)	84 (89.4)	26 (66.7)
Age, median (range) yrs	65 (42–80)	59 (40–80)	65 (58–71) ^a^	60 (35–80)	64 (4–83)
Tumor relapse, n (%)	15 (45.4)	n.a.	34 (47)	44 of 79 (55.7)	21 (46.2)
Tumor stage, n (%):					
I	21 (63.6)	22 (44.0)	32 (44.4)	31 (33.0)	13 (33.3)
II	0	18 (36.0)	24 (33.3)	22 (23.4)	15 (38.5)
III	12 (36.4)	10 (20.0)	16 (22.2)	41 (43.6)	11 (28.2)
Median follow-up (range), months,	24.9 (2–118)	n.a.	47 (40–79) ^b^	n.a.	44 (4–169)
Median RFS, months	n.a.	49.3	n.a.	n.a.	39
5-yrs RFS rate, %	n.a.	n.a.	n.a.	n.a.	44.9
Median DSS, months	n.a.	n.a.	n.a.	n.a.	72
5-yrs DSS rate, %	n.a.	n.a.	57.6	n.a.	57.4
Median OS, months	n.a.	Not reached	n.a.	n.a.	51
5-yrs OS rate, %	51	n.a.	47.4	n.a.	47
Adjuvant therapy, n (%)	n.a.	n.a.	22 (30.5)	75 (79.8)	23 (60.0)
Evaluation of adjuvant treatment	n.a.	n.a.	No correlation between additional chemo-/radiotherapy with RFS and OS	Etoposide-platinum regimen was associated with better outcomes compared to other chemotherapies	No correlation between additional chemo-/radiotherapy with RFS and OS
Potential prognostic factors of RFS	n.a.	n.a.	Tumor size cut-off 3 cm (for systemic recurrence) *	pN	Age (cut-off 65 yrs) and pN
Potential prognostic factors of OS	c-kit expression *	Serum albumin	Not found	Different chemotherapies, T stage, and serum CEA levels	Age (cut-off 65 yrs) and pN

Population analysis-based studies and those including combined LCNEC at histopathological analysis were excluded. Abbreviation: ^a^, interquartile range; ^b^, 95% confident interval; CEA, carcinoembryonic antigen; DSS, disease-specific survival; n.a., not available; yrs, years; OS, overall survival; pN, pathological assessment of the lymph nodes metastasis; RFS, recurrence-free survival (also reported as disease-free survival in some studies); T, tumor stage; *, not confirmed at multivariate analysis.

**Table 2:** Clinical and pathological characteristics of patients and type of treatment.

Parameters	n (%)
N patients	39
Sex: Male Female	26 (66.7%) 13 (33.3%)
BMI: Normal weight Overweight or obesity Not reported	21 (53.8%) 9 (23.1%) 9 (23.1%)
Smoking status: Non-smoker Current or former smoker	5 (12.8%) 34 (87.2%)
Comorbidities: Type 2 diabetes mellitus Hypertension Cardiovascular events	5 (12.8%) 12 (30.8%) 6 (15.4%)
Initial symptoms: Cough Hemoptysis Chest and back pain Fever Asymptomatic	4 (10.3%) 3 (7.7%) 7 (17.9%) 12 (30.8%) 13 (33.3%)
TNM Tumor Stage: I II III	13 (33.3%) 15 (38.5%) 11 (28.2%)
Surgical resection: Wedge resection Lobectomy Bilobectomy Pneumonectomy	3 (7.7%) 27 (69.2%) 2 (5.1%) 7 (18.0%)
Lymph node dissection: Not performed Performed	1 (2.6%) 38 (97.4%)
Adjuvant therapy: Chemotherapy only Radiotherapy only Chemotherapy + radiotherapy No adjuvant therapy	13 (33.3%) 1 (2.6%) 9 (23.0%) 16 (41.0%)

Abbreviation: BMI, body mass index; TNM, tumor, node, and metastasis classification.

**Table 3:** Cox regression univariate and multivariate analysis of prognostic factors influencing recurrence-free survival.

Parameter	n	Univariate RFS		Multivariate RFS	
		*p*	HR (95%CI)	*p*	HR (95%CI)
Sex: M F	26 13	Ref. 0.23	0.54 (0.20–1.48)	-	
Age at diagnosis: <65 years ≥65 years	20 19	Ref. 0.07	2.26 (0.94–5.25)	Ref. 0.008	4.19 (1.46–12.07)
Tumor size: ≤3 cm >3 cm	5 34	Ref. 0.62	1.44 (0.33–6.22)	-	
Pleural invasion: No yes	26 13	Ref. 0.21	1.77 (0.73–4.28)	-	
TNM tumor stage: I II III	13 15 11	0.65 0.99 0.44	Ref. 0.99 (0.34–2.87) 1.54 (0.51–4.61)	-	
pN: N0 N1 N2	18 15 6	0.27 0.67 0.19	Ref. 0.81 (0.31–2.13) 2.19 (0.67–7.20)	0.008 0.65 0.003	Ref. 1.26 (0.46–3.46) 13.56 (2.45–74.89)
Type of surgery: wedge/lobectomy bilobectomy/pneumectomy	30 9	Ref. 0.16	0.41 (0.12–1.40)	Ref. 0.67	0.73 (0.17–3.17)
Adjuvant treatment: no adjuvant therapies chemotherapy or RT alone chemotherapy + RT	16 14 9	0.24 0.15 0.92	Ref. 2.06 (0.77–5.48) 0.94 (0.27–3.23)	0.07 0.06 0.35	Ref. 2.86 (0.96–8.54) 0.51 (0.12–2.11)

In the multivariable model were included variables with a *p*-value less than 0.20 in the univariate analysis. Abbreviation: F, female; M, male; n, number of patients; N0, no lymph node metastasis; N1, metastasis in ipsilateral peribronchial and/or hilar lymph nodes and intrapulmonary nodes; N2, metastasis in ipsilateral mediastinal and/or subcarinal lymph node(s); pN, pathological assessment of the lymph nodes metastasis; RT, radiotherapy; TNM, tumor, node, and metastasis classification; -, not included in the multivariate analysis.

**Table 4:** Cox regression univariate and multivariate analysis of prognostic factors influencing the disease-specific survival.

Parameter	n	Univariate DSS		Multivariate DSS	
		*p*	HR (95%CI)	*p*	HR (95%CI)
Sex: M F	26 13	Ref. 0.31	0.56 (0.18–1.71)	-	
Age at diagnosis: <65 years ≥65 years	20 19	Ref. 0.01	3.91 (1.37–11.20)	Ref. 0.002	9.30 (2.23–38.83)
Tumor size: ≤3 cm >3 cm	5 34	Ref. 0.84	1.17 (0.26–5.19)	-	
Pleural invasion: No yes	26 13	Ref. 0.24	1.78 (0.68–4.71)	-	
TNM tumor stage: I II III	13 15 11	0.27 0.46 0.42	Ref. 0.63 (0.18–2.19) 1.61 (0.51–5.11)	-	
pN: N0 N1 N2	18 15 6	0.04 0.19 0.09	Ref. 0.47 (0.15–1.48) 2.87 (0.84–9.78)	0.003 0.31 0.003	Ref. 0.55 (0.17–1.75) 11.88 (2.28–61.84)
Type of surgery: wedge/lobectomy bilobectomy/pneumectomy	30 9	Ref. 0.21	0.39 (0.09–1.72)	-	
Adjuvant treatment: no adjuvant therapies chemotherapy or RT alone chemotherapy + RT	16 14 9	0.67 0.38 0.76	Ref. 1.66 (0.54–5.07) 1.23 (0.33–4.63)	-	

In the multivariable model were included variables with a *p*-value less than 0.20 in the univariate analysis. Abbreviation: F, female; M, male; n, number of patients; N0, no lymph node metastasis; N1, metastasis in ipsilateral peribronchial and/or hilar lymph nodes and intrapulmonary nodes; N2, metastasis in ipsilateral mediastinal and/or subcarinal lymph node(s); pN, pathological assessment of the lymph nodes metastasis; RT, radiotherapy; TNM, tumor, node, and metastasis classification; -, not included in the multivariate analysis.

## Data Availability

Not applicable.
